# Species distribution and antifungal susceptibility patterns of *Candida* isolates from a public tertiary teaching hospital in the Eastern Cape Province, South Africa

**DOI:** 10.1590/1414-431X20175797

**Published:** 2017-05-15

**Authors:** P. Mnge, B.I. Okeleye, S.D. Vasaikar, T. Apalata

**Affiliations:** 1 Division of Medical Microbiology, Department of Pathology and Laboratory Medicine, Faculty of Health Sciences, Walter Sisulu University, Mthatha, South Africa; 2 National Health Laboratory Services, Mthatha, South Africa; 3 Phytomedicine and Phytopharmacology Research Group, Department of Plant Science, University of the Free State, Phuthaditjhaba, South Africa

**Keywords:** *Candida* species, Distribution, Antifungal susceptibility, Identification, South Africa

## Abstract

*Candida* species are the leading cause of invasive fungal infections, and over the past decade there has been an increased isolation of drug resistant *Candida* species. This study aimed to identify the species distribution of *Candida* isolates and to determine their unique antifungal susceptibility and resistance patterns. During a cross-sectional study, 209 *Candida* isolates (recovered from 206 clinical samples) were collected and their species distribution was determined using ChromAgar *Candida*. The Vitek-2 system (Biomerieux, South Africa) was used to determine minimum inhibitory concentrations (MICs) to azoles (fluconazole, voriconazole), echinocandins (caspofungin, micafungin), polyenes (amphotericin B) and flucytosine. Four species of *Candida* were isolated, of which *C. albicans* was the most frequent, isolated in 45.4% (95/209) of the isolates, followed by *C. glabrata*: 31.1% (65/209). The MICs of the different antifungal drugs varied amongst the species of *Candida*. From the 130 isolates tested for MICs, 90.77% (112/130) were susceptible to all antifungal drugs and 6.9% (9/130) of the isolates were multi-drug resistant. *C. dubliniensis* (n=2) isolates were susceptible to all the above mentioned antifungal drugs. There was no significant difference in species distribution amongst clinical specimens and between patients' genders (P>0.05). An increase in MIC values for fluconazole and flucytosine towards the resistance range was observed. To our knowledge, this is the first report on surveillance of *Candida* species distribution and antifungal susceptibility at a public tertiary teaching hospital in Eastern Cape, South Africa.

## Introduction

*Candida* species are commensal fungi of the human gastrointestinal tract, lower genital tract and mouth cavity. Among immunocompetent individuals, *Candida* species have an inherently low virulence. The incidence of candidiasis is more frequent in immunocompromised patients with impaired physiological and cellular barriers. *Candida* species colonize and invade host tissues wherein they cause localized to invasive systemic infections, which disseminate hematogenously to various organs of the body (1). *Candida* species are the leading cause of mycoses worldwide and the fourth leading cause of invasive nosocomial bloodstream infections with significant crude mortality and morbidity rates (2–5). In South Africa, the incidence and prevalence of *Candida* species is not well documented, however *C. albicans* remains the leading cause of candidiasis worldwide (6–9). Recent epidemiological reports indicate a change in species distribution patterns of *Candida* infections, with an increasing frequency of non-*Albicans Candida* species such as *C. glabrata* and *C. parapsilosis* being isolated from clinical samples (10,11).

In African health care settings, amphotericin B and fluconazole are routinely used to treat *Candida* infections (9,12). The changes in the epidemiology of *Candida* species is in parallel with the emergence of antifungal drug resistant species. Antifungal drug resistance is associated with an uncontrolled distribution and prolonged use of antifungals to treat recurrent infections in immunocompromised patients (13,14). Furthermore, as it is imperative for laboratories to provide identification to the species level, there is continued isolation of new species which are resistant to currently available antifungals.

The growing trend in antifungal drug resistance and emergence of new species of *Candida,* poses a need for regional surveillance of antifungal drug susceptibility profiles, since *in vitro* drug susceptibility patterns are associated with therapeutic outcome. This study sought to determine the distribution and *in vitro* susceptibility profiles of *Candida* species isolated from patients attending Nelson Mandela Academic Complex, a public tertiary teaching hospital in Mthatha, Eastern Cape.

## Material and Methods

### Study population and sampling strategy

A total of 209 *Candida* isolates (from 206 clinical samples) were collected during a cross-sectional study among patients at Nelson Mandela Academic Complex in Mthatha. A standardized data collection form was used to collect information on patients' demographics (age and gender) and clinical history from medical records. Permission to collect patient's clinical data, including laboratory information, was obtained from the hospital and laboratory managers. Ethical clearance was obtained from the Research Ethics Committee of Walter Sisulu University (Ethics Ref. No. 038/13).

### Isolation and identification of *Candida* species

Yeast cells isolated from clinical samples were stored in 10% glycerol (Sigma-Aldrich, South Africa) at –20°C until further use. Isolates were sub-cultured onto freshly prepared Sabaroud dextrose (SAB) agar and incubated overnight at 37°C. The germ tube test was used for the presumptive identification of *Candida* species. Briefly, 24-h fresh cultures were inoculated on 3 drops of human serum and incubated at 37°C. After 2.5 h, the formation of germ tubes was observed under microscopy (40× objective).

The ability of *Candida* species to form differentially colored colonies on chromogenic assay (ChromAgar *Candida*, Media-mage, South Africa) was used to identify *Candida* isolates to the species level. Inocula from 24–48 h SAB-agar cultures were re-cultured onto commercially prepared ChromAgar *Candida* plates and incubated for 48–72 h at 37°C. Intense colony coloration was observed after incubation and species differentiation was done according to the manufacturer’s instructions (14,15).

### Antifungal susceptibility assay

The antifungal susceptibility profile of *Candida* species was determined using the Vitek 2 Systems Version 07.01 (Biomeriex, South Africa) following the CLSI document M27-A3 (2015) (16). The antifungal agents tested were amphotericin B, fluconazole, voriconazole, caspofungin, micafungin and flucytosine. The test was carried out according to the manufacturer's instructions. About 2–3 colonies of 24-h *Candida* cultures were inoculated into 5-mL glass tubes containing 3 mL of 10% saline, adjusted to 2 McFarland standards. Vitek 2 cards with 12-fold serial dilutions of antifungals were placed onto the test tube and loaded onto the Vitek cassette. Loaded cassettes were then placed onto the Vitek instrument and incubated for 9 to 33 h depending on the sample. Standard strains *C. albicans* ATCC 90028 and *C. parapsilosis* ATCC 22019 were used for quality control. The antifungal susceptibility of the isolates was interpreted as sensitive (S), intermediate (I), and resistant (R) according to the CLSI interpretative breakpoints criteria (16,17). The chi-square test was used to test for significant associations between the defined variables, while ANOVA was used to test the difference between groups. SPSS (IMB, USA) version 20.0 for Windows was used for all statistical analyses.

## Results

### Species distribution

A total of 209 isolates of *Candida* were obtained from 206 clinical specimens; the highest number of isolates were from urine specimens (46.5%, n=97), followed by vaginal swabs (30.6%, n=64). The mean age of the patients was 29.7±1.97 years, ranging from 1 month to 87 years. The gender distribution of patients, based on clinical records was 148 (71.9%) females and 46 (22.3%) males. For 5.8% (12/206) isolates, the gender was not stated in clinical records. *C. albicans* accounted for 45.5% (95/209) of the species isolated while 31.1% (65/209) of the species were *C. glabrata,* 12.4% (26/209) *C. tropicalis*, and *C. dubliniensis* accounted for 11.0% (23/209) of the total isolates (Table 1). There was no significant difference in species distribution amongst clinical specimens (X^2^=36; DF=66, and P=0.99) and between patients' genders (X^2^=11.964; DF=22, and P=0.958).

**Table 1.** Distribution of Candida species among clinical specimens (n=209).

Specimen	*C. albicans*	*C. glabrata*	*C. tropicalis*	*C. dubliniensis*	n	Percentage (%)
Urine	44	32	12	9	97	46.5%
Vaginal swab	24	19	11	10	64	30.6%
Sputum	14	6	1	2	23	11.0%
Blood culture	10	5	1	0	16	7.7%
Pus	2	0	0	1	3	1.4%
Unknown	1	1	1	0	3	1.4%
Ascetic fluid	0	2	0	1	3	1.4%

### Antifungal susceptibility testing

The minimum inhibitory concentrations (MICs) and antifungal susceptibility of *Candida* species to the various antifungal drugs are summarized in Tables 2 and 3. The results are presented by species as cumulative counts of susceptible organisms at each concentration throughout the full dilution series.

**Table 2.** Minimum inhibitory concentrations (MIC) of clinical isolates of Candida (n=130) to various antifungal drugs.

Antifungal	MIC range (μg/mL)*										
	0.065	0.125	0.25	0.5	1	2	4	8	16	32	64
Fluconazole											
*C. albicans*					84	4	4	10	1		6
*C. glabrata*					5		3	1			2
*C. tropicalis*					5			2			2
*C. dubliniensis*					2						
Voriconazole											
*C. albicans*		96	4	1	4	1	1	2			
*C. glabrata*		9					2				
*C. tropicalis*		7						1	1		
*C. dubliniensis*		2									
Caspofungin											
*C. albicans*			102		3	4	4				
*C. glabrata*			9				2				
*C. tropicalis*			6				2	1			
*C. dubliniensis*			2								
Micafungin											
*C. albicans*	101	2		2			4				
*C. glabrata*	6	3					2				
*C. tropicalis*	6	1					2				
*C. dubliniensis*	2										
Amphotericin B											
*C. albicans*			72	31	2		4				
*C. glabrata*			3	6					2		
*C. tropicalis*			6	1					2		
*C. dubliniensis*			2								
Flucytosine											
*C. albicans*					103			2	2		2
*C. glabrata*					7	3				1	
*C. tropicalis*					6	1	1				1
*C. dubliniensis*					2						

* MIC breakpoints' interpretation: fluconazole susceptible: MIC ≤8 µg/mL; fluconazole intermediate: MIC 16-32 µg/mL; fluconazole resistant: MIC ≥64 µg/mL; micafungin and caspofungin susceptible: MIC ≤0.25; micafungin and caspofungin intermediate: no clinical breakpoint; micafungin and caspofungin resistant: MIC ≥1 µg/mL; flucytosine susceptible: MIC ≤4 µg/mL; flucytosine intermediate: MIC 8-16 µg/mL; flucytosine resistant: MIC ≥32 µg/mL; voriconazole susceptible: MIC ≤1 µg/mL; voriconazole intermediate: MIC 2 µg/mL; voriconazole resistant: MIC≥ 4 µg/mL; amphotericin B susceptible: MIC ≤1 µg/mL; amphotericin B Intermediate: no clinical breakpoint; amphotericin B resistant: MIC >1 µg/mL.

**Table 3.** Antifungal susceptibility patterns of *Candida* species to different antifungal drugs.

Antifungal	Species (n=130)				
	C. albicans (n=109)	*C. glabrata* (n=11)	*C. tropicalis* (n=8)	*C. dubliniensis* (n=2)	P
Fluconazole					0.277
S	102 (93.6%)	9 (81.8%)	6 (75%)	2 (100%)	
I	2 (1.8%)				
R	5 (4.6%)	2 (18.2%)	2 (25%)		
Voriconazole					0.061
S	105 (96.3%)	9 (81.8%)	6 (75%)	2 (100%)	
I	1 (0.9%)				
R	3 (2.8%)	2 (18.2%)	2 (25%)		
Caspofungin					0.065
S	105 (96.3%)	9 (81.8%)	6 (75%)	2 (100%)	
R	4 (3.7%)	2 (18.2%)	2 (25%)		
Micafungin					0.068
S	105 (96.3%)	9 (81.8%)	6 (75%)	2 (100%)	
R	4 (3.7%)	2 (18.2%)	2 (25%)		
Amphotericin B					0.068
S	105 (96.3%)	9 (81.8%)	6 (75%)	2 (100%)	
R	4 (3.7%)	2 (18.2%)	2 (25%)		
Flucytosine					0.0934
S	102 (93.6%)	10 (90.9%)	7 (87.5%)	2 (100%)	
I	2 (1.8%)				
R	5 (4.6%)	1 (9.1%)	1 (12.5%)		

S: susceptible; I: intermediate; R: resistant. P<0.05 (chi-square test).

For *C. albicans,* resistance to fluconazole was 4.6% (5/109 at MIC ≥64 μg/mL), voriconazole resistance was 2.8% (3/109, MIC ≥8 μg/mL), caspofungin and micafungin resistance were 3.7% (4/109 at MIC ≥4 μg/mL). In addition, voriconazole resistance was 2.8% (3/109, MIC ≥8 μg/mL) and flucytosine resistance was 3.7% (4/109, MIC ≥32 μg/mL). Intermediate resistance to flucytosine (MIC=8 μg/mL) and fluconazole (MIC=16 μg/mL) was observed in 2 isolates of *C. albicans* and 1 other isolate exhibited intermediate resistance to voriconazole at MIC of 2 μg/mL.

A similar resistance pattern was observed in 2 isolates of both *C. glabrata* and *C. tropicalis*. One isolate from each species was fully resistant to all antifungal drugs, while the other isolate was resistant to all antifungal drugs except for flucytosine at MIC ≥64 μg/mL in *C. tropicalis* and MIC of 32 μg/mL in *C. glabrata*. Notably, at flucytosine MIC of ≤1 μg/mL (susceptible), 3 (2.6%) *C. albicans* and 2 (18.2%) *C. glabrata* isolates were resistant to all other classes of antifungals. Two isolates of *C. albicans* were resistant to all antifungals, except flucytosine (MIC ≤16 μg/mL) and intermediate to voriconazole at MIC of 2 μg/mL.

## Discussion

The study sought to compare the species distribution pattern of *Candida* and to compare the antifungal susceptibility patterns of 6 antifungal drugs against the isolated species of *Candida*. The most common species isolated was *C. albicans*, and this finding is similar to previously published reports in South Africa, where *C. albicans* was the most commonly isolated species from clinical samples (9,12). In a study that determined the microbial carriage in bloodstream infections, *C. albicans* was responsible for 31% (21/68) of all fungal infections (18). The findings of this study also correlate with reports from the Neonatal Intensive Care Unit in Dr. George Mukhari Hospital, South Africa, where C. *albicans* was the most common species isolated at the unit in 2002 (9). A recent report by Makhado et al. (9) has, however, indicated a shift in the epidemiology of candidiasis with *C. krusei* replacing C. *albicans* as the most common species isolated. Although *C. albicans* was the most common species isolated in this present study, the rate of non-albicans *Candida* species varied amongst the clinical specimens and these species were isolated in more than 50% of the clinical samples.

In contrast to *C. albicans* being the most commonly isolated species, *C. glabrata, C. krusei* and *C. tropicalis* are also reported to be leading causes of candidiasis. The frequency of these non-albicans *Candida* species varies throughout the world. This variation may be a result of the underlying medical condition, geographic distribution and patients' age and gender (1,19–21). *C. glabrata* and *C. tropicalis* are the most common species isolated in neutropenic patients, in catheter related infections, and in adults, while *C. krusei* has been reported as the leading cause of candidemia. The increase in isolation of non-albicans *Candida* species is in parallel with a decrease in the isolation of *C. albicans*. (1,14,18,20)

The high frequency of *Candida* species isolated from vaginal and urine samples from female patients can be explained by the imbalance in vaginal microflora as a result of diabetes and vaginal estrogenization, which in turns gives rise to vulvovaginal candidiasis (21,22). Symptomatic vulvovaginal candidiasis (VVC) occurs in women aged 18–84 years and is associated with a significant morbidity rate. Approximately 75% of women experience one episode of VVC in their lifetime (23–25). The underlying medical conditions for the isolates were vaginal discharge, retroviral disease and pelvic inflammatory discharge. On most urine samples, the clinical diagnosis was stated as illegible thus making it difficult to ascertain if the presence of yeast in urine represents a true infection or merely colonization and contamination of the bladder (26).

The ongoing change in the epidemiology of *Candida* species is in parallel with the emergence of antifungal drug resistant *Candida* species (14). This is especially true for fluconazole, which is used as the first line drug treatment for hematological malignancy, HIV/AIDS and oropharyngeal candidiasis in South Africa and Africa as a whole. This, in turn, leads to the selection of less susceptible isolates, with an inherent or acquired resistance to fluconazole, especially *C. albicans* and *C. tropicalis* isolates (8,9,12,13,27). The widespread use of fluconazole does not only lead to selection of antifungal drug resistant species, but also to a shift from *C. albicans* as the leading cause of candidiasis to non-albicans species such as *C. glabrata* and *C. tropicalis* as the causative agents of *Candida* infections (14,28).

Relatively low levels of fluconazole resistance were observed in all species of *Candida* isolated; it is worth mentioning that the non-*albicans* species had the highest levels of resistance compared to *C. albicans*. Primary resistance to fluconazole is reported in C. *albicans*, C. *tropicalis* and C. *krusei* isolates. Multi-drug resistance was observed in *C. albicans*, *C. glabrata* and *C. tropicalis* isolates, indicating a major public health concern and reflecting the inappropriate use of antifungals drugs. This also supports the importance of ongoing *in vitro* surveillance and careful monitoring of antifungal treatment regimens since *in vitro* resistance is associated with therapeutic failure (12,14,29).

The lowest MIC (0.065 μg/mL) in all species of *Candida* was observed in micafungin (Table 2); this echinocandin class of antifungals is said to confer excellent fungicidal effects against *Candida* species. Echinocandins are not routinely used in antifungal therapy due to their hepatic effect. Flucytosine resistance, more especially in *C. glabrata* isolates, is reported when the drug is administered as monotherapy. It is, therefore, suggested that in order to achieve optimal flucytosine activity, the drug should be administered in combination with amphotericin B, and/or any azole and echinocandins (30–32).

The antifungal susceptibility of echinocandins and liposomal amphotericin B against *Candida* biofilms was investigated by Marcos-Zambrano (33). It was found that *C. tropicalis* biofilms had the highest level of resistance and amphotericin B was unable to reduce the metabolic activity of the biofilms. In a study conducted by Blignaut et al. (34), the South African clade of *C. albicans* had an 8.4% level of resistance to amphotericin B and the clade was found to be naturally resistant to that drug. That finding is supported in this study, in which *C. tropicalis* isolates had a 25% level of resistance compared to *C. albicans* and *C. glabrata.* Although the results are statistically not significant, this finding has to be considered when amphotericin B is used to treat *C. tropicalis* infections. This is equally important, since amphotericin B is routinely used to treat fluconazole-resistant infections (33).

There was a degree of variability in the MIC values within the same class of antifungals, for example the MIC range for micafungin was between 0.025 to 4 μg/mL and caspofungin MIC ranged from 0.065 to 8 μg/mL. On the other hand, MIC values for fluconazole ranged from 1 to 64 μg/mL and for voriconazole from 0.125 to 16 μg/mL. A similar pattern was also observed by Villareal et al. (35) in *C. glabrata* isolates. The differences in MIC values within species and classes are indicative of species-specific resistance patterns. Hence, there is a need for regional surveillance of fungal species distribution, and antifungal therapeutic regimes should be implemented based on epidemiological data and antifungal sensitivity. The low MICs and increased spectrum of activity for voriconazole and micafungin in *C. albicans* and non-*albicans* species suggest good clinical activity for these drugs.

The epidemiology of *Candida* infections in Africa, a home for new and emerging drug-resistant *Candida* species, is not well documented (9,14), and this study presented the first regional surveillance in the Eastern Cape province to investigate the prevalence and antifungal susceptibility patterns of *Candida* isolates.
